# Synthesis and structure of *trans*-bis­(4-amino-3-nitro­benzoato-κ*O*)bis­(4-amino-3-nitro­benzoic acid-κ*O*)di­aqua­manganese(II) dihydrate

**DOI:** 10.1107/S2414314624000403

**Published:** 2024-01-19

**Authors:** Feruza S. Narmanova, Khayit Kh. Turaev, Aziz B. Ibragimov, Jamshid M. Ashurov

**Affiliations:** a Termez State University, Barkamol Avlod Street 43, Termez city, Uzbekistan; b Institute of General and Inorganic Chemistry of Uzbekistan Academy of Sciences, 100170, Mirzo Ulug’bek str., 77a, Uzbekistan; c Institute of Bioorganic Chemistry, Academy of Sciences of Uzbekistan, 100125, M. Ulugbek Str 83, Tashkent, Uzbekistan; Howard University, USA

**Keywords:** 4-amino 3-nitro­benzoic acid, Mn^II^, crystal structure, hydrogen bond

## Abstract

The manganese title complex, [Mn(C_7_H_5_N_2_O_4_)_2_(C_7_H_6_N_2_O_4_)_2_(H_2_O)_2_]·2H_2_O, is one of the first 4-amino 3-nitro­benzoic acid (4 A3NBA) monoligand metal complexes to be synthesized. It crystallizes in the centrosymmetric monoclinic space group *P*2_1_/*n* with the complex mol­ecules located on inversion centers. Four 4 A3NBA ligand mol­ecules are monodentately coordinated by the Mn^2+^ ion through the carb­oxy­lic oxygen atoms while the other two positions of the inner coordination sphere are occupied by water mol­ecules, giving rise to a distorted octa­hedron, and two water mol­ecules are in the outer coordination sphere. There are two intra­molecular hydrogen bonds in the complex mol­ecule. The first is of the common N—H⋯O=N type, while the second is a rarely occurring very strong hydrogen bond in which a common proton is shared by two uncoordinated oxygen atoms of neighboring carboxyl­ate groups. In the crystal, an intricate system of inter­molecular hydrogen bonds links the complex mol­ecules into a three-dimensional-network.

## Abstract 2

The reaction of 4-amino 3-nitro­benzoic acid and manganese dichloride tetra­hydrate in an ethanol–water mixture yielded the title complex[Mn(C_7_H_5_N_2_O_4_)_2_(C_7_H_6_N_2_O_4_)_2_(H_2_O)_2_]·2H_2_O. In the crystal, mol­ecules are linked by N—H⋯O, O—H⋯O and C—H⋯O hydrogen bonds.

## Graphical abstract

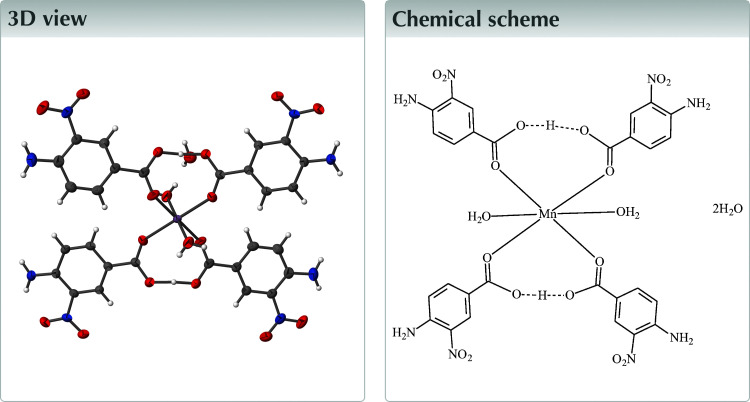

## Structure description

**Figure 1:**
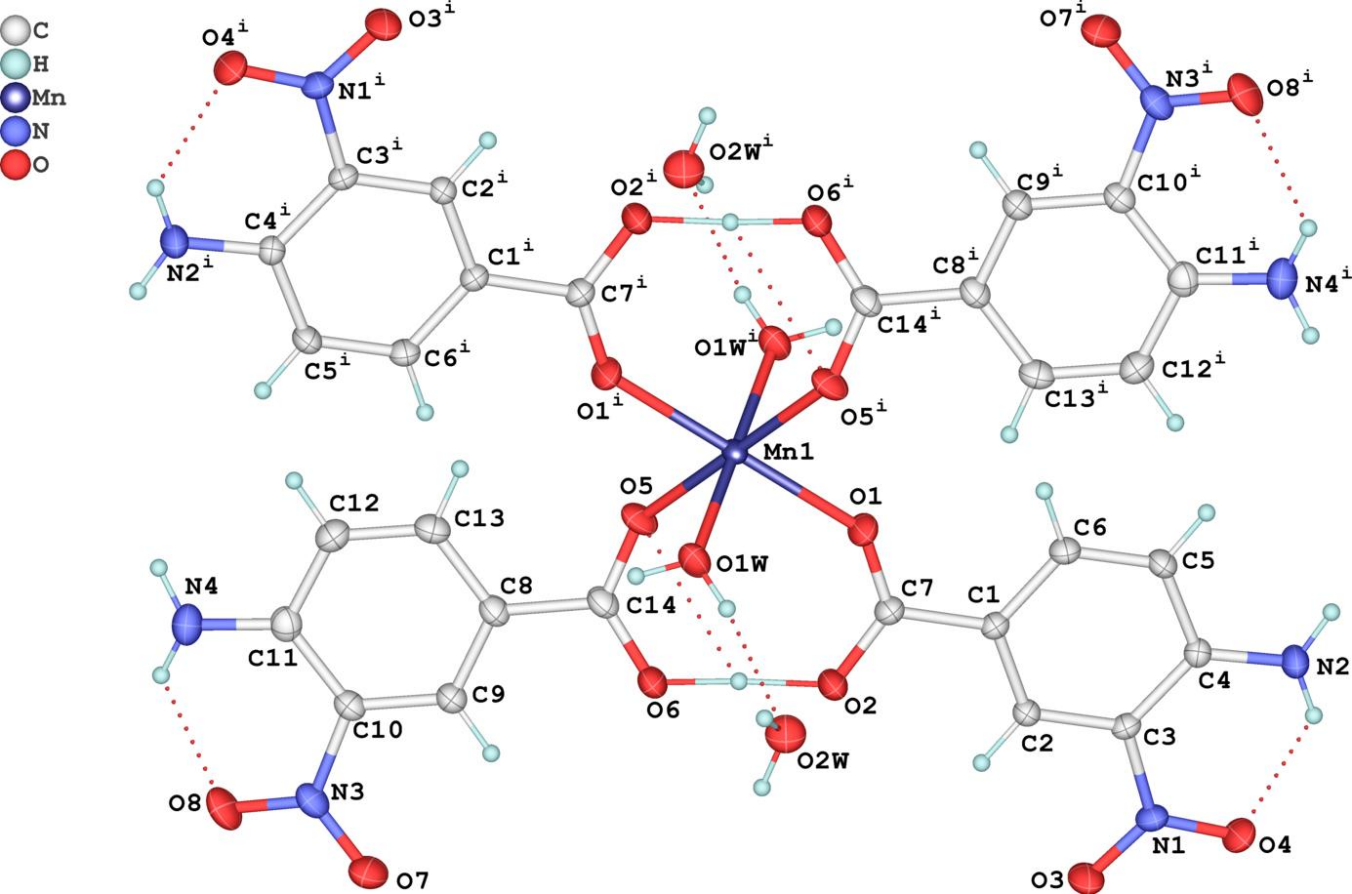
The mol­ecular structure of the title compound, showing the atom-labeling scheme and displacement ellipsoids drawn at the 50% probability level. H atoms are shown as small spheres of arbitrary radius and hydrogen bonds are shown as dashed lines. Symmetry code: (i) 1 − *x*, 1 − *y*, −*z*.

The mol­ecular structure of the title complex is shown in Fig. 1 ▸. It crystallizes in the centrosymmetric monoclinic space group *P*2_1_/*n* with the complex mol­ecules located on inversion centers. Four 4-amino 3-nitro­benzoic acid (4 A3NBA) ligands are monodentately coordinated by the Mn^2+^ ion through the oxygen atoms of carb­oxy­lic groups while two other positions of the inner coordination sphere are occupied by water mol­ecules. The outer coordination sphere contains two water mol­ecules, *i.e.* the complex is crystal hydrate. The length of the Mn—O1 bond is 2.1575 (12) Å while Mn—O5 is 2.1600 (13) Å and Mn—O1*W* = 2.1630 (14) Å and bond angles are in the range 84.29 (5) to 95.71 (5)°. The geometry of the manganese atom is therefore a slightly distorted octa­hedron. The carboxyl­ate groups C7,O2,O1 and C14,O6,O5 are practically coplanar with the aromatic rings to which they are attached, forming dihedral angles of 4.1 (1) and 11.9 (1)°, respectively. The analogous angles for nitro groups N1,O3,O4 and N3,O7,O8 are 2.82 (9) and 8.6 (1)°. Thus in the ligand with the C8–C13 aromatic ring, the functional groups are more inclined relatively to the benzene ring.

There are two intra­molecular hydrogen bonds in the complex mol­ecule (Table 1 ▸). The first bond is of the usual N—H⋯O=N type, closing a six-membered ring with an 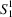 (6) graph-set motif (Etter 1990 ▸; Ibragimov *et al.*, 2017 ▸; Ruzmetov *et al.*, 2022 ▸). The second is a rarely occurring very strong hydrogen bond closing a nine-membered ring where a common proton, H20, is shared by two uncoordinated oxygen atoms O2 and O6 of neighboring carboxyl­ate groups. The atom H20, situated between the two oxygen atoms, is located closer to atom O2 at a distance of 1.270 (2) Å [and 1.198 (2) Å from O6]. Despite this, it is impossible to indicate which of the four carb­oxy­lic groups present are deprotonated. The total negative charge of the carb­oxy­lic groups is 2 and it compensates the +2 charge of the Mn^2+^ ion.

**Figure 2:**
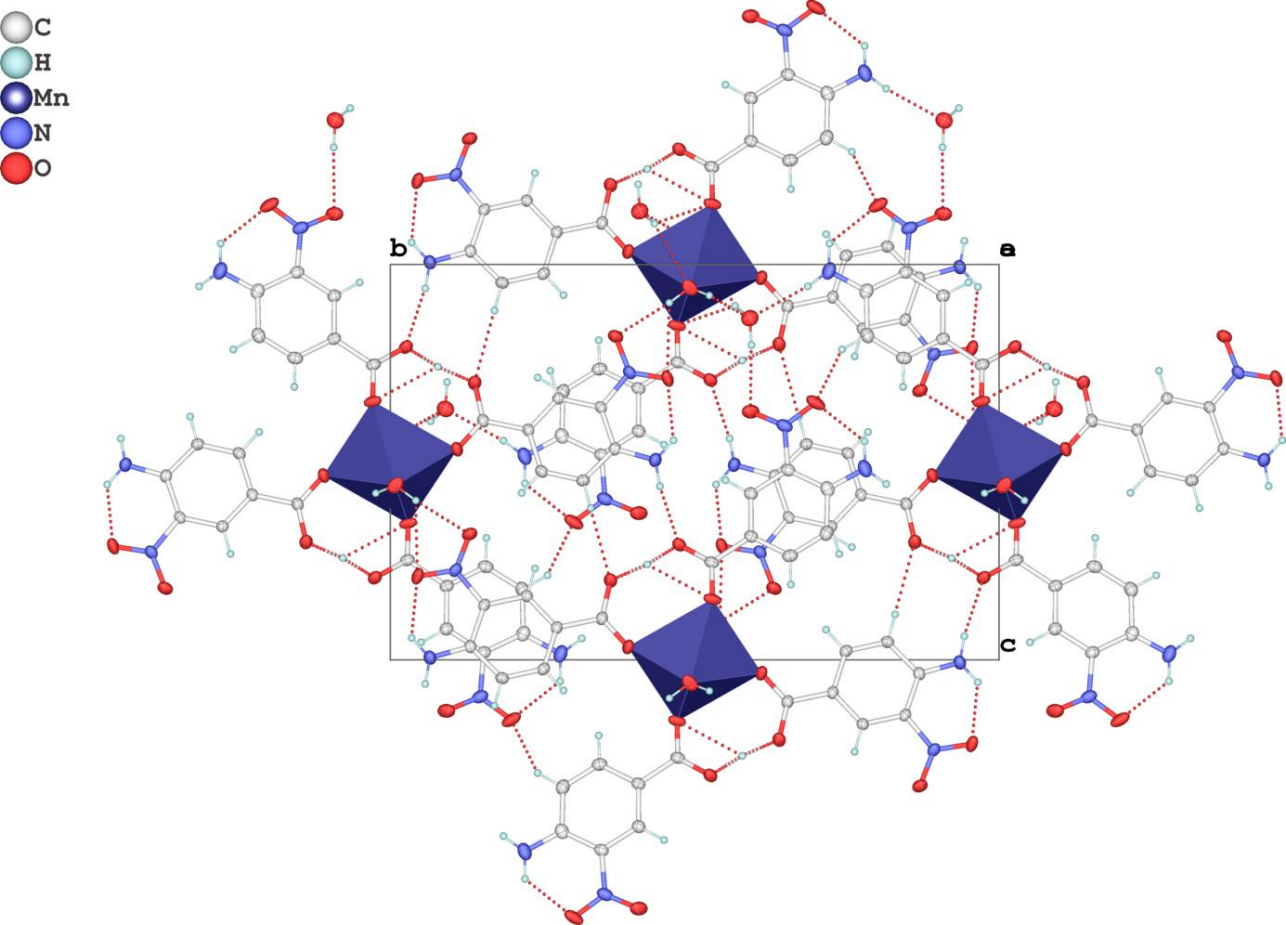
The crystal packing viewed along [100] showing the O—H⋯O, N—H⋯O and C—H⋯O hydrogen bonds (dashed red lines) in the crystal structure.

There are 17 proton-acceptor oxygen atoms, 4 proton-donor nitro­gen atoms and 2 water mol­ecules in the title complex. These atoms are involved in a complex system of inter­molecular hydrogen bonds (Table 1 ▸). Moreover, three weak C—H⋯O hydrogen bonds are also observed in the structure (Table 1 ▸, Fig. 2 ▸). Together these hydrogen bonds link the complex mol­ecules into a three-dimensional network (Fig. 2 ▸.).

## Synthesis and crystallization

All reagents and solvents were purchased from Sigma-Aldrich (Darmstadt, Germany) and they were used as received. MnCl_2_·H_2_O (0.198 g, 1.0 mmol) was dissolved in a small amount of water. 4-Amino 3-nitro­benzoic acid (0.364 g, 2 mmol) was dissolved in a mixed solvent of 3 ml of absolute alcohol and 3 ml of distilled water. After dropwise addition of the 4 A3NBA solution to the manganese salt solution, the resultant solution was stirred for 2 h with a magnetic stirrer at 55°C. The solution was allowed to stand at 30°C in a beaker with small holes in the cover for evaporation. After about eight days, block-shaped single crystals ofthe title compound appeared. Analysis calculated: C_28_H_30_MnN_8_O_20_: C, 39.40%; H, 3.54; N, 13.13%. Found: C, 39.32%; H, 3.47%; N, 13.08%.

## Refinement

Crystal data, data collection and structure refinement details for the structure of the synthesized compound are summarized in Table 2 ▸.

## Supplementary Material

Crystal structure: contains datablock(s) I. DOI: 10.1107/S2414314624000403/bv4050sup1.cif

Structure factors: contains datablock(s) I. DOI: 10.1107/S2414314624000403/bv4050Isup2.hkl

CCDC reference: 2324651

Additional supporting information:  crystallographic information; 3D view; checkCIF report

## Figures and Tables

**Table 1:** Hydrogen-bond geometry (Å, °)

*D*—H⋯*A*	*D*—H	H⋯*A*	*D*⋯*A*	*D*—H⋯*A*
O1*W*—H1*WA*⋯O3^i^	0.83 (2)	2.06 (2)	2.8850 (19)	176 (2)
O1*W*—H1*WA*⋯O4^i^	0.83 (2)	2.55 (2)	3.1418 (19)	130 (2)
O1*W*—H1*WA*⋯N1^i^	0.83 (2)	2.63 (2)	3.4029 (19)	157 (2)
O1*W*—H1*WB*⋯O2*W*	0.81 (2)	1.98 (2)	2.784 (2)	170 (2)
O2—H2*O*⋯O5	1.27 (4)	2.57 (4)	3.448 (2)	124 (2)
O6—H2*O*⋯O2	1.20 (4)	1.27 (4)	2.4541 (18)	168 (4)
N2—H2*A*⋯O6^ii^	0.86	2.19	3.0160 (19)	162
N2—H2*B*⋯O4	0.85	1.99	2.629 (2)	131
N4—H4*A*⋯O8	0.87	2.03	2.648 (2)	128
N4—H4*B*⋯O2*W* ^i^	0.87	2.16	3.021 (2)	173
C5—H5⋯O2^ii^	0.93	2.57	3.489 (2)	170
C9—H9⋯N2^iii^	0.93	2.66	3.544 (2)	160
C12—H12⋯O8^iv^	0.93	2.42	3.178 (2)	138
O2*W*—H2*WA*⋯O7^v^	0.86	2.14	2.995 (2)	173
O2*W*—H2*WB*⋯O1*W* ^vi^	0.85	2.39	3.127 (2)	145
O2*W*—H2*WB*⋯O5^vii^	0.85	2.65	3.341 (2)	139

Symmetry codes: (i) 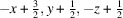 ; (ii) 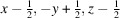 ; (iii) 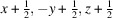 ; (iv) 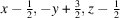 ; (v) 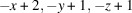 ; (vi) 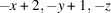 ; (vii) 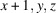 .

**Table 2:** Experimental details

Crystal data	
Chemical formula	[Mn(C_7_H_5_MnN_2_O_4_)_2_(C_7_H_6_MnN_2_O_4_)_2_(H_2_O)_2_]·2H_2_O
*M*_r_	853.54
Crystal system, space group	Monoclinic, *P*2_1_/*n*
Temperature (K)	293
*a*, *b*, *c* (Å)	7.0419 (1), 19.2513 (3), 12.7175 (2)
β (°)	100.513 (2)
*V* (Å^3^)	1695.12 (5)
*Z*	2
Radiation type	Cu *K*α
μ (mm^−1^)	4.08
Crystal size (mm)	0.28 × 0.22 × 0.14

Data collection	
Diffractometer	XtaLAB Synergy, Single source at home/near, HyPix3000
Absorption correction	Multi-scan (*CrysAlis PRO*; Rigaku OD, 2023 ▸)
*T*_min_, *T*_max_	0.523, 1.000
No. of measured, independent and observed [*I* > 2σ(*I*)] reflections	3291, 3291, 2966
*R*_int_	0.037
(sin θ/λ)_max_ (Å^−1^)	0.615

Refinement	
*R*[*F* ^2^ > 2σ(*F* ^2^)], *wR*(*F* ^2^), *S*	0.035, 0.097, 1.06
No. of reflections	3291
No. of parameters	269
No. of restraints	3
H-atom treatment	H atoms treated by a mixture of independent and constrained refinement
Δρ_max_, Δρ_min_ (e Å^−3^)	0.27, −0.44

Computer programs: *CrysAlis PRO* (Rigaku OD, 2023 ▸), *SHELXT* (Sheldrick, 2015*a* ▸), *SHELXL2018/3* (Sheldrick, 2015*b* ▸) and *OLEX2* (Dolomanov *et al.*, 2009 ▸).

## full crystallographic data

### Crystal data

**Table:**

[Mn(C_7_H_5_MnN_2_O_4_)_2_(C_7_H_6_MnN_2_O_4_)_2_(H_2_O)_2_]·2H_2_O	*F*(000) = 878
*M**_r_* = 853.54	*D*_x_ = 1.672 Mg m^−^^3^
Monoclinic, *P*2_1_/*n*	Cu *K*α radiation, λ = 1.54184 Å
*a* = 7.0419 (1) Å	Cell parameters from 11377 reflections
*b* = 19.2513 (3) Å	θ = 2.3–71.4°
*c* = 12.7175 (2) Å	µ = 4.08 mm^−^^1^
β = 100.513 (2)°	*T* = 293 K
*V* = 1695.12 (5) Å^3^	Block, light pink
*Z* = 2	0.28 × 0.22 × 0.14 mm

### Data collection

**Table:**

XtaLAB Synergy, Single source at home/near, HyPix3000 diffractometer	3291 independent reflections
Radiation source: micro-focus sealed X-ray tube	2966 reflections with *I* > 2σ(*I*)
Detector resolution: 10.0000 pixels mm^-1^	*R*_int_ = 0.037
ω scans	θ_max_ = 71.5°, θ_min_ = 4.2°
Absorption correction: multi-scan (CrysAlisPro; Rigaku OD, 2023)	*h* = −8→8
*T*_min_ = 0.523, *T*_max_ = 1.000	*k* = −23→22
3291 measured reflections	*l* = −15→15

### Refinement

**Table:**

Refinement on *F*^2^	Primary atom site location: dual
Least-squares matrix: full	Secondary atom site location: difference Fourier map
*R*[*F*^2^ > 2σ(*F*^2^)] = 0.035	Hydrogen site location: mixed
*wR*(*F*^2^) = 0.097	H atoms treated by a mixture of independent and constrained refinement
*S* = 1.06	*w* = 1/[σ^2^(*F*_o_^2^) + (0.0608*P*)^2^ + 0.2227*P*] where *P* = (*F*_o_^2^ + 2*F*_c_^2^)/3
3291 reflections	(Δ/σ)_max_ < 0.001
269 parameters	Δρ_max_ = 0.27 e Å^−^^3^
3 restraints	Δρ_min_ = −0.44 e Å^−^^3^

### Special details

**Table:**

Geometry. All esds (except the esd in the dihedral angle between two l.s. planes) are estimated using the full covariance matrix. The cell esds are taken into account individually in the estimation of esds in distances, angles and torsion angles; correlations between esds in cell parameters are only used when they are defined by crystal symmetry. An approximate (isotropic) treatment of cell esds is used for estimating esds involving l.s. planes.
Refinement. The hydrogen atoms of water molecules and amino groups were located in difference-Fourier maps and refined freely. The H atoms of the benzene ring were calculated geometrically with C—H = 0.93 A° and *U*_iso_(H) = 1.2*U*_eq_(C).

### Fractional atomic coordinates and isotropic or equivalent isotropic displacement parameters (Å^2^)

**Table:**

	*x*	*y*	*z*	*U*_iso_*/*U*_eq_	
Mn1	0.500000	0.500000	0.000000	0.02947 (13)	
O1	0.4809 (2)	0.39058 (6)	0.03322 (11)	0.0489 (4)	
O1W	0.8075 (2)	0.50832 (7)	0.05836 (12)	0.0464 (3)	
H1WA	0.848 (4)	0.5418 (9)	0.0964 (19)	0.070*	
H1WB	0.879 (4)	0.4760 (9)	0.078 (2)	0.070*	
O2	0.6022 (2)	0.36153 (7)	0.20147 (10)	0.0515 (4)	
H2O	0.619 (6)	0.422 (2)	0.242 (3)	0.155 (15)*	
O3	0.5562 (2)	0.13009 (7)	0.31842 (10)	0.0560 (4)	
O4	0.4652 (2)	0.04466 (7)	0.21207 (11)	0.0545 (4)	
O5	0.4355 (2)	0.52907 (8)	0.15374 (10)	0.0457 (3)	
O6	0.6122 (2)	0.47449 (7)	0.29174 (10)	0.0483 (4)	
O7	0.7667 (2)	0.59190 (8)	0.62838 (11)	0.0590 (4)	
O8	0.6914 (2)	0.69874 (8)	0.65088 (11)	0.0612 (4)	
N1	0.4964 (2)	0.10712 (7)	0.22733 (11)	0.0363 (3)	
N2	0.3472 (2)	0.06488 (7)	0.00606 (12)	0.0431 (4)	
H2A	0.302809	0.057204	−0.060340	0.052*	
H2B	0.365807	0.034904	0.056060	0.052*	
N3	0.6948 (2)	0.64740 (8)	0.59270 (12)	0.0410 (4)	
N4	0.5654 (3)	0.77954 (8)	0.48424 (14)	0.0481 (4)	
H4A	0.587279	0.778868	0.553450	0.058*	
H4B	0.516269	0.814368	0.445160	0.058*	
C1	0.4755 (2)	0.27328 (8)	0.08203 (13)	0.0297 (3)	
C2	0.5071 (2)	0.22409 (8)	0.16193 (13)	0.0294 (3)	
H2	0.557893	0.237429	0.231674	0.035*	
C3	0.4636 (2)	0.15439 (8)	0.13907 (12)	0.0291 (3)	
C4	0.3884 (2)	0.13131 (8)	0.03426 (12)	0.0298 (3)	
C5	0.3551 (3)	0.18352 (9)	−0.04571 (13)	0.0351 (4)	
H5	0.303735	0.170966	−0.115751	0.042*	
C6	0.3963 (2)	0.25158 (9)	−0.02257 (13)	0.0333 (4)	
H6	0.371609	0.284412	−0.076992	0.040*	
C7	0.5212 (2)	0.34754 (8)	0.10562 (13)	0.0335 (4)	
C8	0.5352 (2)	0.59261 (9)	0.31259 (13)	0.0330 (4)	
C9	0.6043 (2)	0.59219 (9)	0.42108 (13)	0.0338 (4)	
H9	0.643858	0.550494	0.455105	0.041*	
C10	0.6160 (2)	0.65326 (9)	0.48065 (13)	0.0333 (4)	
C11	0.5581 (2)	0.71818 (9)	0.43305 (15)	0.0358 (4)	
C12	0.4899 (3)	0.71660 (9)	0.32115 (15)	0.0409 (4)	
H12	0.453293	0.758055	0.285614	0.049*	
C13	0.4760 (3)	0.65655 (10)	0.26390 (14)	0.0384 (4)	
H13	0.426366	0.657803	0.190964	0.046*	
C14	0.5253 (3)	0.52789 (9)	0.24730 (13)	0.0352 (4)	
O2W	1.0872 (2)	0.40951 (8)	0.13520 (11)	0.0542 (4)	
H2WA	1.119577	0.406931	0.203299	0.081*	
H2WB	1.161577	0.433931	0.103999	0.081*	

### Atomic displacement parameters (Å^2^)

**Table:**

	*U*^11^	*U*^22^	*U*^33^	*U*^12^	*U*^13^	*U*^23^
Mn1	0.0436 (2)	0.02138 (19)	0.01972 (19)	−0.00214 (14)	−0.00417 (15)	0.00137 (12)
O1	0.0807 (10)	0.0235 (6)	0.0358 (7)	−0.0052 (6)	−0.0070 (6)	0.0050 (5)
O1W	0.0456 (7)	0.0419 (8)	0.0444 (8)	0.0030 (6)	−0.0114 (6)	−0.0121 (6)
O2	0.0808 (10)	0.0297 (7)	0.0340 (7)	−0.0014 (6)	−0.0157 (6)	−0.0019 (5)
O3	0.0926 (11)	0.0393 (7)	0.0283 (7)	−0.0025 (7)	−0.0099 (7)	0.0063 (5)
O4	0.0851 (10)	0.0251 (6)	0.0454 (8)	−0.0059 (6)	−0.0086 (7)	0.0091 (5)
O5	0.0584 (8)	0.0528 (8)	0.0221 (6)	0.0066 (6)	−0.0030 (5)	−0.0078 (5)
O6	0.0748 (9)	0.0322 (7)	0.0303 (7)	0.0027 (6)	−0.0106 (6)	−0.0053 (5)
O7	0.0864 (11)	0.0519 (9)	0.0318 (7)	0.0122 (8)	−0.0079 (7)	0.0002 (6)
O8	0.0824 (11)	0.0605 (9)	0.0366 (8)	0.0046 (8)	−0.0006 (7)	−0.0225 (7)
N1	0.0459 (8)	0.0285 (7)	0.0305 (7)	0.0019 (6)	−0.0038 (6)	0.0066 (5)
N2	0.0643 (10)	0.0265 (7)	0.0336 (8)	−0.0027 (7)	−0.0044 (7)	−0.0028 (6)
N3	0.0479 (9)	0.0443 (9)	0.0287 (7)	−0.0013 (7)	0.0012 (6)	−0.0084 (6)
N4	0.0589 (10)	0.0334 (8)	0.0510 (10)	0.0015 (7)	0.0072 (8)	−0.0093 (7)
C1	0.0353 (8)	0.0235 (8)	0.0280 (8)	0.0022 (6)	0.0001 (6)	0.0019 (6)
C2	0.0360 (8)	0.0249 (8)	0.0247 (7)	0.0012 (6)	−0.0010 (6)	−0.0008 (6)
C3	0.0347 (8)	0.0242 (8)	0.0267 (8)	0.0035 (6)	0.0011 (6)	0.0042 (6)
C4	0.0333 (8)	0.0243 (7)	0.0299 (8)	0.0013 (6)	0.0003 (6)	−0.0020 (6)
C5	0.0468 (9)	0.0304 (9)	0.0244 (8)	−0.0012 (7)	−0.0036 (7)	−0.0015 (6)
C6	0.0422 (9)	0.0282 (8)	0.0268 (8)	0.0010 (7)	−0.0013 (7)	0.0045 (6)
C7	0.0421 (9)	0.0248 (8)	0.0307 (8)	0.0003 (7)	−0.0012 (7)	0.0030 (6)
C8	0.0391 (9)	0.0335 (9)	0.0252 (8)	−0.0017 (7)	0.0030 (6)	−0.0033 (6)
C9	0.0394 (9)	0.0312 (8)	0.0286 (8)	0.0011 (7)	0.0004 (7)	−0.0015 (6)
C10	0.0362 (8)	0.0354 (9)	0.0264 (8)	−0.0002 (7)	0.0012 (7)	−0.0027 (6)
C11	0.0331 (8)	0.0337 (9)	0.0402 (9)	−0.0015 (7)	0.0058 (7)	−0.0043 (7)
C12	0.0484 (10)	0.0324 (9)	0.0401 (10)	0.0039 (8)	0.0032 (8)	0.0050 (7)
C13	0.0437 (9)	0.0418 (10)	0.0271 (8)	0.0016 (8)	−0.0005 (7)	0.0029 (7)
C14	0.0432 (9)	0.0372 (9)	0.0237 (8)	−0.0032 (7)	0.0021 (7)	−0.0037 (7)
O2W	0.0659 (9)	0.0496 (8)	0.0448 (8)	0.0015 (7)	0.0040 (7)	0.0036 (6)

### Geometric parameters (Å, º)

**Table:**

Mn1—O1^i^	2.1575 (12)	N4—H4A	0.8655
Mn1—O1	2.1575 (12)	N4—H4B	0.8680
Mn1—O5^i^	2.1600 (13)	C1—C2	1.377 (2)
Mn1—O5	2.1600 (13)	C1—C6	1.409 (2)
Mn1—O1W	2.1630 (14)	C1—C7	1.484 (2)
Mn1—O1W^i^	2.1630 (14)	C2—C3	1.395 (2)
O1—C7	1.233 (2)	C2—H2	0.9300
O1W—H1WA	0.827 (16)	C3—C4	1.413 (2)
O1W—H1WB	0.813 (16)	C4—C5	1.419 (2)
O2—C7	1.277 (2)	C5—C6	1.363 (2)
O2—H2O	1.27 (4)	C5—H5	0.9300
O3—N1	1.2398 (19)	C6—H6	0.9300
O4—N1	1.2314 (19)	C8—C9	1.377 (2)
O5—C14	1.242 (2)	C8—C13	1.406 (2)
O6—C14	1.275 (2)	C8—C14	1.492 (2)
O6—H2O	1.20 (4)	C9—C10	1.393 (2)
O7—N3	1.233 (2)	C9—H9	0.9300
O8—N3	1.237 (2)	C10—C11	1.416 (2)
N1—C3	1.4307 (19)	C11—C12	1.417 (3)
N2—C4	1.345 (2)	C12—C13	1.360 (3)
N2—H2A	0.8581	C12—H12	0.9300
N2—H2B	0.8510	C13—H13	0.9300
N3—C10	1.436 (2)	O2W—H2WA	0.8558
N4—C11	1.345 (2)	O2W—H2WB	0.8537
			
O1^i^—Mn1—O1	180.0	C3—C2—H2	119.7
O1^i^—Mn1—O5^i^	92.57 (6)	C2—C3—C4	121.96 (14)
O1—Mn1—O5^i^	87.43 (6)	C2—C3—N1	116.77 (14)
O1^i^—Mn1—O5	87.43 (6)	C4—C3—N1	121.27 (14)
O1—Mn1—O5	92.57 (6)	N2—C4—C3	125.16 (15)
O5^i^—Mn1—O5	180.0	N2—C4—C5	118.87 (14)
O1^i^—Mn1—O1W	84.29 (5)	C3—C4—C5	115.97 (14)
O1—Mn1—O1W	95.71 (5)	C6—C5—C4	121.70 (15)
O5^i^—Mn1—O1W	88.12 (5)	C6—C5—H5	119.1
O5—Mn1—O1W	91.88 (5)	C4—C5—H5	119.1
O1^i^—Mn1—O1W^i^	95.71 (5)	C5—C6—C1	121.42 (15)
O1—Mn1—O1W^i^	84.29 (5)	C5—C6—H6	119.3
O5^i^—Mn1—O1W^i^	91.88 (5)	C1—C6—H6	119.3
O5—Mn1—O1W^i^	88.12 (5)	O1—C7—O2	124.97 (16)
O1W—Mn1—O1W^i^	180.0	O1—C7—C1	118.99 (15)
C7—O1—Mn1	142.04 (12)	O2—C7—C1	116.03 (14)
Mn1—O1W—H1WA	118.6 (17)	C9—C8—C13	117.94 (15)
Mn1—O1W—H1WB	125.3 (18)	C9—C8—C14	121.66 (15)
H1WA—O1W—H1WB	106 (2)	C13—C8—C14	120.40 (15)
C7—O2—H2O	124.8 (18)	C8—C9—C10	121.00 (16)
C14—O5—Mn1	135.11 (13)	C8—C9—H9	119.5
C14—O6—H2O	120.4 (19)	C10—C9—H9	119.5
O4—N1—O3	121.07 (14)	C9—C10—C11	121.91 (15)
O4—N1—C3	119.93 (14)	C9—C10—N3	116.57 (15)
O3—N1—C3	119.00 (14)	C11—C10—N3	121.50 (15)
C4—N2—H2A	116.6	N4—C11—C10	125.85 (17)
C4—N2—H2B	116.7	N4—C11—C12	118.74 (17)
H2A—N2—H2B	126.7	C10—C11—C12	115.41 (15)
O7—N3—O8	121.58 (15)	C13—C12—C11	122.26 (16)
O7—N3—C10	119.44 (14)	C13—C12—H12	118.9
O8—N3—C10	118.98 (16)	C11—C12—H12	118.9
C11—N4—H4A	117.6	C12—C13—C8	121.45 (16)
C11—N4—H4B	115.2	C12—C13—H13	119.3
H4A—N4—H4B	124.9	C8—C13—H13	119.3
C2—C1—C6	118.40 (14)	O5—C14—O6	123.99 (16)
C2—C1—C7	120.90 (14)	O5—C14—C8	118.82 (16)
C6—C1—C7	120.70 (14)	O6—C14—C8	117.19 (15)
C1—C2—C3	120.53 (15)	H2WA—O2W—H2WB	115.4
C1—C2—H2	119.7		
			
C6—C1—C2—C3	0.7 (2)	C13—C8—C9—C10	−0.2 (3)
C7—C1—C2—C3	179.94 (15)	C14—C8—C9—C10	178.81 (16)
C1—C2—C3—C4	0.8 (3)	C8—C9—C10—C11	−0.1 (3)
C1—C2—C3—N1	−178.46 (15)	C8—C9—C10—N3	−178.47 (16)
O4—N1—C3—C2	−178.27 (16)	O7—N3—C10—C9	7.9 (3)
O3—N1—C3—C2	1.7 (2)	O8—N3—C10—C9	−173.23 (17)
O4—N1—C3—C4	2.5 (2)	O7—N3—C10—C11	−170.52 (17)
O3—N1—C3—C4	−177.55 (16)	O8—N3—C10—C11	8.4 (3)
C2—C3—C4—N2	178.46 (16)	C9—C10—C11—N4	−179.64 (18)
N1—C3—C4—N2	−2.3 (3)	N3—C10—C11—N4	−1.3 (3)
C2—C3—C4—C5	−1.7 (2)	C9—C10—C11—C12	−0.6 (3)
N1—C3—C4—C5	177.57 (15)	N3—C10—C11—C12	177.70 (16)
N2—C4—C5—C6	−179.08 (17)	N4—C11—C12—C13	−179.19 (18)
C3—C4—C5—C6	1.0 (3)	C10—C11—C12—C13	1.7 (3)
C4—C5—C6—C1	0.5 (3)	C11—C12—C13—C8	−2.1 (3)
C2—C1—C6—C5	−1.4 (3)	C9—C8—C13—C12	1.3 (3)
C7—C1—C6—C5	179.42 (17)	C14—C8—C13—C12	−177.73 (17)
Mn1—O1—C7—O2	0.8 (3)	Mn1—O5—C14—O6	−51.2 (3)
Mn1—O1—C7—C1	−178.67 (15)	Mn1—O5—C14—C8	128.20 (16)
C2—C1—C7—O1	−175.97 (17)	C9—C8—C14—O5	169.25 (17)
C6—C1—C7—O1	3.2 (3)	C13—C8—C14—O5	−11.7 (3)
C2—C1—C7—O2	4.5 (3)	C9—C8—C14—O6	−11.3 (3)
C6—C1—C7—O2	−176.31 (16)	C13—C8—C14—O6	167.69 (17)

Symmetry code: (i) −*x*+1, −*y*+1, −*z*.

### Hydrogen-bond geometry (Å, º)

**Table:**

*D*—H···*A*	*D*—H	H···*A*	*D*···*A*	*D*—H···*A*
O1*W*—H1*WA*···O3^ii^	0.83 (2)	2.06 (2)	2.8850 (19)	176 (2)
O1*W*—H1*WA*···O4^ii^	0.83 (2)	2.55 (2)	3.1418 (19)	130 (2)
O1*W*—H1*WA*···N1^ii^	0.83 (2)	2.63 (2)	3.4029 (19)	157 (2)
O1*W*—H1*WB*···O2*W*	0.81 (2)	1.98 (2)	2.784 (2)	170 (2)
O2—H2*O*···O5	1.27 (4)	2.57 (4)	3.448 (2)	124 (2)
O6—H2*O*···O2	1.20 (4)	1.27 (4)	2.4541 (18)	168 (4)
N2—H2*A*···O6^iii^	0.86	2.19	3.0160 (19)	162
N2—H2*B*···O4	0.85	1.99	2.629 (2)	131
N4—H4*A*···O8	0.87	2.03	2.648 (2)	128
N4—H4*B*···O2*W*^ii^	0.87	2.16	3.021 (2)	173
C5—H5···O2^iii^	0.93	2.57	3.489 (2)	170
C9—H9···N2^iv^	0.93	2.66	3.544 (2)	160
C12—H12···O8^v^	0.93	2.42	3.178 (2)	138
O2*W*—H2*WA*···O7^vi^	0.86	2.14	2.995 (2)	173
O2*W*—H2*WB*···O1*W*^vii^	0.85	2.39	3.127 (2)	145
O2*W*—H2*WB*···O5^viii^	0.85	2.65	3.341 (2)	139

Symmetry codes: (ii) −*x*+3/2, *y*+1/2, −*z*+1/2; (iii) *x*−1/2, −*y*+1/2, *z*−1/2; (iv) *x*+1/2, −*y*+1/2, *z*+1/2; (v) *x*−1/2, −*y*+3/2, *z*−1/2; (vi) −*x*+2, −*y*+1, −*z*+1; (vii) −*x*+2, −*y*+1, −*z*; (viii) *x*+1, *y*, *z*.

## References

[bb1] Dolomanov, O. V., Bourhis, L. J., Gildea, R. J., Howard, J. A. K. & Puschmann, H. (2009). *J. Appl. Cryst.* **42**, 339–341.

[bb2] Etter, M. C. (1990). *Acc. Chem. Res.* **23**, 120–126.

[bb3] Ibragimov, A. B., Ashurov, Z. M. & Zakirov, B. S. (2017). *J. Struct. Chem.* **58**, 588–590.

[bb4] Rigaku OD (2023). *CrysAlis PRO*. Rigaku Oxford Diffraction Ltd, Yarnton, England.

[bb5] Ruzmetov, A., Ibragimov, A., Ashurov, J., Boltaeva, Z., Ibragimov, B. & Usmanov, S. (2022). *Acta Cryst.* E**78**, 660–664. 10.1107/S205698902200531X PMC9431793 36072132

[bb6] Sheldrick, G. M. (2015*a*). *Acta Cryst.* A**71**, 3–8.

[bb7] Sheldrick, G. M. (2015*b*). *Acta Cryst.* C**71**, 3–8.

